# Correction to “Mifepristone increases mRNA translation rate, triggers the unfolded protein response, increases autophagic flux, and kills ovarian cancer cells in combination with proteasome or lysosome inhibitors”

**DOI:** 10.1002/1878-0261.70270

**Published:** 2026-05-19

L. Zhang, M. B. Hapon, A. A. Goyeneche, R. Srinivasan, C. D. Gamarra‐Luques, E. A. Callegari, et al. “Mifepristone increases mRNA translation rate, triggers the unfolded protein response, increases autophagic flux, and kills ovarian cancer cells in combination with proteasome or lysosome inhibitors,” *Molecular Oncology* 10, no. 7 (2016): 1099–1117, https://doi.org/10.1016/j.molonc.2016.05.001.

In Figure 5D, the authors acknowledge that the blot originally presented as the GAPDH protein loading control was incorrect. A corrected image of the GAPDH loading control for all proteins shown in Figure 5D is provided below. The authors confirm that the Figure 5 legend, as well as all experimental results and the corresponding conclusions reported in the paper, remain unaffected.

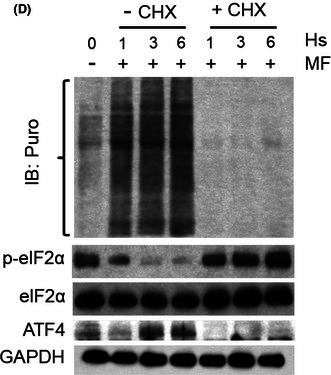

Corrected Figure 5D.

The authors apologize for this error and for any inconvenience caused.

